# Arctiin targets oxidative stress and inflammation, restores Neuregulin-1, and improves neurobehavioral outcomes in neonatal hypoxic-ischemic brain injury

**DOI:** 10.1016/j.crphar.2026.100253

**Published:** 2026-02-17

**Authors:** Skandar Babak, Tahereh Safari, Hamed Fanaei

**Affiliations:** a Department of Physiology, School of Medicine, Zahedan University of Medical Sciences, Zahedan, Iran; b Pharmacology Research Center, Zahedan University of Medical Sciences, Zahedan, Iran; c Cellular and Molecular Research Center, Research Institute of Cellular and Molecular Sciences in Infectious Diseases, Zahedan University of Medical Sciences, Zahedan, Iran

**Keywords:** Arctiin, Ischemia, Neuroprotection, Neuregulin-1, Antioxidant, Inflammation, BDNF

## Abstract

**Objective:**

Hypoxic-ischemic brain damage (HIBD) represents a major cause of neonatal morbidity and mortality, resulting from perinatal oxygen deprivation and impaired cerebral blood flow. This study aims to investigate the neuroprotective effects of Arctiin, a bioactive lignan derived from *Arctium lappa*, recognized for its potent antioxidant and anti-inflammatory properties, in a neonatal rat model of HIBD.

**Materials and methods:**

Neonatal rats at postnatal day 8 were randomly assigned to four groups: Sham-operated (SHAM), Hypoxia-Ischemia (HI), Hypoxia-Ischmia with Solvent control (HI/SO), and Hypoxia-Ischemia treated with Arctiin (HI/Arc). HIBD was induced via unilateral carotid artery ligation followed by exposure to hypoxia. The HI/Arc group was administered Arctiin orally at a dosage of 60 mg/kg daily for seven consecutive days. Behavioral performance, biochemical parameters, histological integrity, and gene expression profiles were assessed to evaluate the neuroprotective efficacy of Arctiin.

**Results:**

Arctiin administration resulted in a significant reduction in C-reactive protein (CRP), and total oxidant capacity (TOC). Simultaneously, it enhanced total antioxidant capacity (TAC) and brain-derived neurotrophic factor (BDNF) levels. Histological analysis showed diminished infarct volume in the Arctiin-treated group. Moreover, gene expression studies revealed significant restoration of Neuregulin-1 (NRG-1) in group treated by arctiin. Neurobehavioral assessments further confirmed significant improvements in sensorimotor function in the Arctiin-treated group.

**Conclusion:**

Our study provides evidence indicating that Arctiin mitigates hypoxic-ischemic brain damage in rat pups through a synergistic mechanism involving the suppression of inflammation and oxidative stress, coupled with the upregulation of critical neuroprotective genes and proteins, specifically NRG-1 gene expression and BDNF protein levels. Future studies should investigate the precise molecular pathways downstream of NRG-1 that mediate Arctiin's neuroprotective effects.

## Highlights

• Arctiin shows neuroprotection in a neonatal rat model of hypoxic-ischemic brain injury.
• It reduces key markers of inflammation and oxidative stress post-injury.
• Treatment boosts antioxidant capacity levels.
• Arctiin diminishes brain infarct volume and restores neuroprotective Neuregulin-1.
• It leads to significant improvement in neurobehavioral and sensorimotor outcomes.

## Graphical abstract

**Figure:**
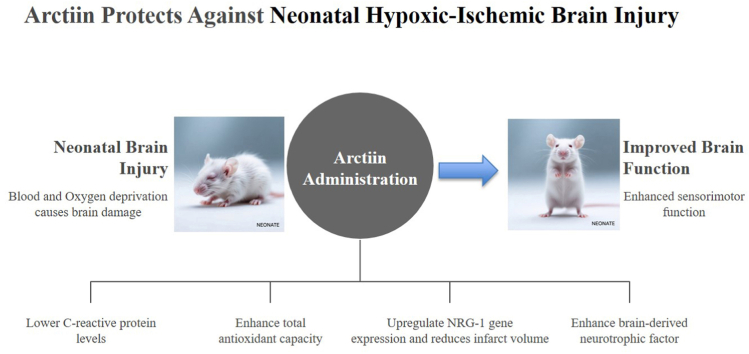

## 1 Introduction

Neonatal hypoxic-ischemic brain damage (HIBD) is a leading cause of mortality and permanent neurological disability (Bornavard and Fanaei, 2020; Lan et al., 2019). While therapeutic hypothermia has improved outcomes, it remains the sole approved intervention and has significant limitations, including a narrow therapeutic window (within 6 h of birth), variable efficacy, and incompletely understood long-term benefits (Ceri and Ozdemir, 2025). Moreover, as a physical intervention, it does not directly target the multifactorial molecular cascades—such as oxidative stress and neuroinflammtion that propagate injury in the hours to days following the initial insult (Ceri and Ozdemir, 2025; Sabir, 2025). The lack of effective, targeted pharmacological adjuncts represents a critical gap in clinical management (Sabir, 2025). This complex pathogenesis, characterized by intertwined cascades of oxidative stress and inflammation that lead to neuronal death, underscores the urgent need for novel neuroprotective agents capable of modulating these specific pathways (Ceri and Ozdemir, 2025; Sabir, 2025).

The search for effective pharmacological adjuncts has increasingly turned to naturally derived bioactive compounds (Mohsenpour et al., 2021; Reyes-Corral et al., 2021). This strategic focus is justified by several key advantages: many natural products possess inherently multi-target mechanisms of action, aligning with the need to address the interconnected pathways of HIBD (Mohsenpour et al., 2021; Reyes-Corral et al., 2021). Their long historical use in ethno medical traditions often provides preliminary insights into their safety and bioactivity. Furthermore, their diverse chemical scaffolds offer unique opportunities for modulating complex biological systems, such as the neuroinflammatory and oxidative stress responses central to hypoxic-ischemic injury (Mohsenpour et al., 2021; Reyes-Corral et al., 2021). These characteristics position natural products as a valuable source for discovering novel neuroprotectants capable of modulating the multi-factorial pathology of neonatal HIBD (Mohsenpour et al., 2021; Reyes-Corral et al., 2021).

While several compounds with antioxidant and anti-inflammatory properties show promise in preclinical models—such as *trans*-piceatannol, which ameliorates hypoxia-ischemia by modulating similar pathways (Al-Jaber et al., 2024; Chen and Cheang, 2024)—the exploration of safe, naturally derived agents with strong translational potential remains critical. Arctiin, a major bioactive lignan from *Arctium lappa* (burdock), represents a compelling candidate due to its established dual anti-inflammatory and antioxidant activities, its history of use in traditional medicine, and its favorable pharmacokinetic profile for oral administration (Gao et al., 2018). Preclinical evidence suggests arctiin modulates key signaling pathways (e.g., PI3K/AKT, NF-κB) implicated in HIBD (Gao et al., 2018) (Yosri et al., 2023; Shyam and Sabina, 2024). However, its mechanisms of action in the context of *neonatal* HIBD, particularly concerning critical neurotrophic and neuroprotective factors, are not well defined.

This study was therefore designed to evaluate the therapeutic potential of orally administered Arctiin in a validated neonatal rat model of HIBD. We specifically hypothesized that Arctiin would improve neurobehavioral outcomes by simultaneously mitigating oxidative stress and inflammation while upregulating protective signaling. To test this, we investigated a defined post-injury treatment regimen and assessed a comprehensive set of outcomes: histological damage (infarct volume), key molecular markers (systemic oxidative stress, antioxidant capacity, inflammation, brain-derived neurotrophic factor (BDNF), the expression of the crucial neuroprotective gene Neuregulin-1 (NRG-1), and sensorimotor function. By focusing on this multi-target approach and highlighting the novel investigation of NRG-1 in Arctiin's mechanism, our work aims to provide a robust preclinical foundation for Arctiin as a potential therapeutic intervention for neonatal HIBD.

## 2 Materials and Methods

### 2.1 Animals and experimental groups

Throughout the study, the experimental protocol followed ethical guidelines for laboratory animal use, as approved by the Ethics Committee of Zahedan University of Medical Sciences (IR.ZAUMS.AEC.1402.002). Arctiin (≥95% purity, CAS, 20362-31-6) was sourced from Sigma-Aldrich Co. Ltd (St. Louis, USA). In this study, 20 female Wistar rats weighing between 200 and 220 g were selected from the Laboratory Animal Research Center of Zahedan University of Medical Sciences. To acclimate the animals to the environment, they were kept under controlled environmental conditions in the center for one week before the experiment began. During this period, the animals had access to food and water, and the laboratory maintained a 12-h light/dark cycle with a temperature of ±22 °C. To induce pregnancy, two females and one male rat were housed together in a cage. A vaginal smear was collected daily from the females to detect the presence of sperm. After a positive sperm test, the female rats were transferred to a separate cage.

After delivery, the rat pups were divided into four groups.

• The group receiving Arctiin was treated with 60 mg/kg Arctiin (Liu et al., 2021) for seven consecutive days following the hypoxic-ischemic insult via gavage.

Following birth, the pups were randomly assigned into 4 groups of 16.

1. **Sham Group (SHAM)**: Surgery was performed, but cerebral blood flow was not interrupted, and hypoxia was not induced.
2. **Hypoxia-Ischemia Group (HI)**: Animals in this group underwent surgery, with the right common carotid artery being permanently ligated, followed by exposure to 8% oxygen for 90 min.
3. **Hypoxia-Ischemia + Solvent Group (HI/SOLVENT)**: In this group, surgery was performed, the right common carotid artery was ligated, hypoxia was induced, and the animals received the DMSO solvent.
4. **Hypoxia-Ischemia + Arctiin Group (HI/Arctiin)**: These animals underwent surgery with the right common carotid artery ligation and were exposed to 8% oxygen for 90 min. Additionally, they received Arctiin (60 mg/kg) for 7 days.

### 2.2 Hypoxia-ischemia induction method

Hypoxia-ischemia was induced using the Rice-Vannucci method (132). On the eighth day after birth, the pups were anesthetized with ketamine (100 mg/kg) and xylazine (10 mg/kg). A longitudinal incision was made along the midline of the cervical skin, and the right common carotid artery (CCA) was permanently ligated using 0/6 nylon sutures. One hour after recovery, the pups were exposed to 8% oxygen for 90 min (Khayat et al., 2023). Seven days after hypoxia-ischemia, initial neurological-behavioral assessments were performed. Afterward, brain tissue was collected to evaluate brain edema, infarct area size, inflammatory markers, and oxidative stress.

### 2.3 Neurological impairment assessment methods

Eight brains were used for biochemical analysis, and another eight were used for histological evaluation (brain edema and infarct volume).

### 2.4 Cliff avoidance test

The cliff avoidance test was performed on 15-day-old pups to assess the integrity of sensory inputs and motor outputs. The pups were placed at the edge of a platform (30 cm × 30 cm × 30 cm) with their chest on the edge, and the time to retreat or move away from the platform's edge was recorded. If a pup fell from the platform or failed to respond within 60 s, a 60-s response time was assigned (Khayat et al., 2023).

### 2.5 Negative geotaxis test

The negative geotaxis test was used to evaluate the sensory-motor performance of the neonatal rats. The pups were placed on a rough surface inclined at a 30° angle, with their heads facing downward. The time taken for the pups to rotate 180° towards the top was recorded. The maximum time allowed for this task was 90 s (Khayat et al., 2023).

### 2.6 Brain edema and infarct volume assessment methods

After behavioral testing, the animals were euthanized by ketamine (100 mg/kg) and xylazine (10 mg/kg), and the brains were removed. Cross-sections of the brain (1 mm thick) were obtained using a rat brain matrix and immersed in 2% triphenyltetrazolium chloride (TTC) solution (Khayat et al., 2023).

Images of the sections were captured using a camera, and the area of the ischemic region was measured using ImageJ software. The following formula was used to calculate the ischemic area volume (Bornavard et al., 2020):

$$\text{Volume}\;\text{of}\;\text{ischemic}\;\text{region}=\left(\frac{\text{volume}\;\text{of}\;\text{contralateral}\;\text{hemisphere}-\text{the}\;\text{volume}\;\text{of}\;\text{the}\;\text{nonischemic}\;\text{region}\;\text{of}\;\text{the}\;\text{ipsilateral}\;\text{hemisphere}}{\text{volume}\;\text{of}\;\text{contralateral}\;\text{hemisphere}}\;\right)\times 100$$

The following formula was used to measure brain edema (Bornavard et al., 2020):

$$\text{Edema}=\frac{\left(\text{volume}\;\text{of}\;\text{ipsilateral}\;\text{hemisphere}-\text{volume}\;\text{of}\;\text{contralateral}\;\text{hemisphere}\right)}{\text{volume}\;\text{of}\;\text{contralateral}\;\text{hemisphere}}$$

### 2.7 Measurement of inflammatory factors, oxidative stress, and BDNF levels

On day 15 after birth, following behavioral analysis, the pups were deeply anesthetized using ketamine (100 mg/kg) and xylazine (10 mg/kg), and their brain tissues were rapidly extracted. The right hemisphere was separated, homogenized in ice-cold phosphate-buffered saline (PBS; 1:10 w/v), and centrifuged at 3500×*g* for 20 min at 4 °C. The supernatant was collected and stored at −70 °C for subsequent analysis. The levels of BDNF and CRP were quantified using specific commercial enzyme-linked immunosorbent assay (ELISA) kits according to the manufacturers' instructions. To assess systemic oxidative stress, the Total Oxidant Status (TOS) and Total Antioxidant Capacity (TAC) of the brain homogenates were determined using established colorimetric methods (Ahmed, 2025).

### 2.8 Extraction of mRNA and quantitative real-time polymerase chain reaction (qRT-PCR)

The cerebral cortex was carefully removed from the pups under anesthesia, which was induced using Ketamine (100 mg/kg) and Xylazine (10 mg/kg). The collected tissue samples were then stored at −80 °C for further analysis. To assess the expression levels of NRG-1, the brain tissue was homogenized, and total RNA was isolated using the Total RNA Extraction Kit (Parstous, Iran). The quality and quantity of the extracted RNA were determined with the Thermo Scientific NanoDrop 2000 spectrophotometer (ThermoFisher Scientific Inc., USA). The RNA was subsequently reverse-transcribed into cDNA using the cDNA Synthesis Kit (Parstous, Iran). Quantitative PCR (qPCR) was performed using the following thermal cycling conditions: an initial denaturation step at 95 °C for 15 min, followed by 45 cycles of denaturation at 95 °C for 30 s, annealing at 61 °C for 30 s, and extension at 72 °C for 30 s. Real-time PCR was carried out on the CFX 96 Real-Time System (Bio-Rad Laboratories Inc., California, USA). The β-actin gene served as the reference gene for data normalization (Table 1).

**Table 1:** Presents the primer sequences used in the study.

Gene	Forward Sequence	Reverse Sequence	Product size (bp)
NRG-1	5′-GCGATAAAGGGACAGCGTCA-3′	3′-ATCCTGTGTTGTTGGGCTGG-5′	252
β-actin	5′-AAGTCCCTCACCCTCCCAAAAG-3′	3′-AAGCAATGCTGTCACCTTCCC-5′	98

### 2.9 Data analysis methods

Data were analyzed using GraphPad Prism software version 8. Statistical analysis was performed using one-way ANOVA followed by Bonferroni's post hoc test. Data are presented as mean ± standard error of the mean (Mean ± SEM). A p-value <0.05 was considered statistically significant.

## 3 Results

Our study results showed that Arctiin administration in pups significantly reduced the damage induced by hypoxic-ischemic (HI) brain injury. Arctiin treatment resulted in decreased levels of C-reactive protein (CRP) and oxidative stress markers (TOS). On the other hand, Arctiin increased protective factors such as total antioxidant capacity (TAC) and brain-derived neurotrophic factor (BDNF) against HI. Ultimately, brain edema, infarct volume, and neurological behavior scores were significantly improved in the Arctiin group compared to the control group.

### 3.1 Determination and comparison of NRG1 mRNA expression in ischemic brain tissue

**Fig. 1:**
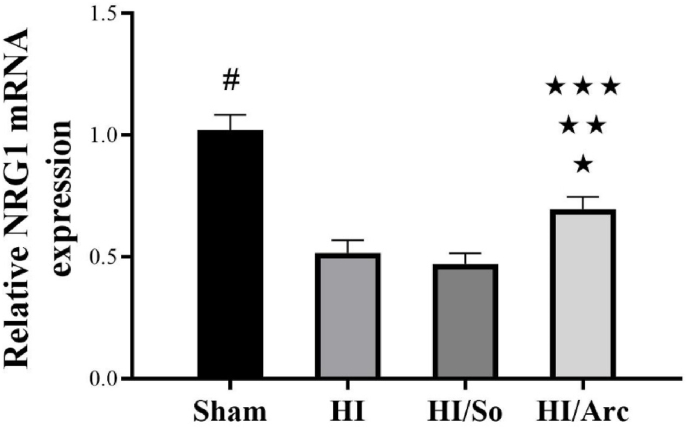
Relative NRG1 mRNA expression levels in the Sham, HI (hypoxia-ischemia), HI/So, and HI/Arc groups (n = 8). Data are presented as mean ± SEM. ∗p = 0.0008, HI vs. HI/Arc ∗∗p = 0.0021 HI/So vs. HI/Arc ∗∗∗p = 0.0027 Sham vs. HI/Arc # p < 0.0001Sham vs. HI and HI/So.

The results of the relative NRG1 mRNA expression analysis are presented in Fig. 1. The data indicate significant differences between the experimental groups. The Sham group served as the control, showing baseline expression levels. HI and HI/So groups exhibited a marked decrease in NRG1 mRNA expression compared to the Sham group (p < 0.0001). HI/Arc group, treated with Arctiin, showed a significant increase in NRG1 mRNA expression compared to the HI/So group (p = 0.029), suggesting a potential therapeutic effect.

### 3.2 Determination and comparison of CRP levels in ischemic brain tissue

**Fig. 2:**
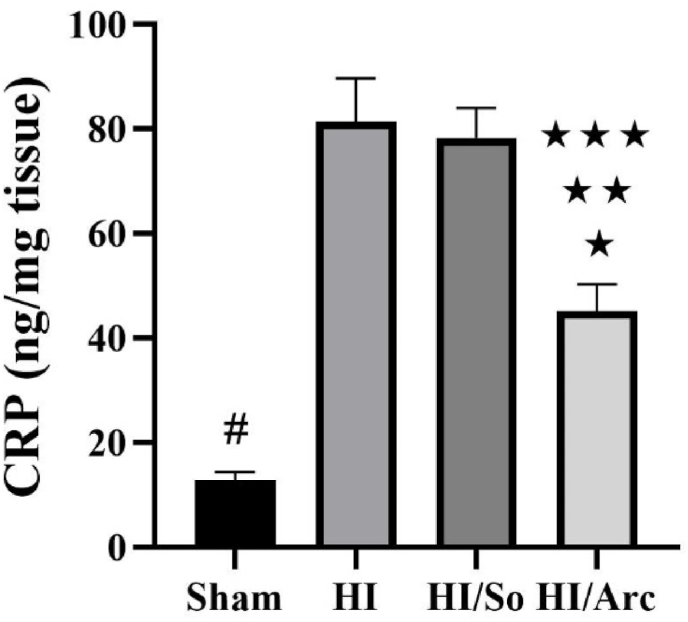
C-reactive protein (CRP) levels in the Sham, HI, HI/So, and HI/Arc groups (n = 8). Data are presented as mean ± SEM. ∗p = 0.0008 HI vs. HI/Arc ∗∗p = 0.0021 HI/So vs. HI/Arc ∗∗∗p = 0.0027 Sham vs. HI/Arc # p < 0.0001 Sham vs. HI and HI/So.

As shown in Fig. 2, the mean CRP level in the HI + Arctiin group (45.1 ± 5.19 nmol/mg tissue) was significantly lower compared to the HI/So (78.2 ± 5.79 nmol/mg tissue, p = 0.0021) and HI groups (81.37 ± 8.35 nmol/mg tissue, p = 0.0008). Additionally, the mean CRP level in the HI/Arctiin group was significantly higher compared to the Sham group (12.75 ± 1.63, p = 0.0027). The mean CRP level in the Sham group was significantly lower than in the HI/So and HI groups (p < 0.0001).

### 3.3 Determination and comparison of TAC and TOS levels in ischemic brain tissue

**Fig. 3:**
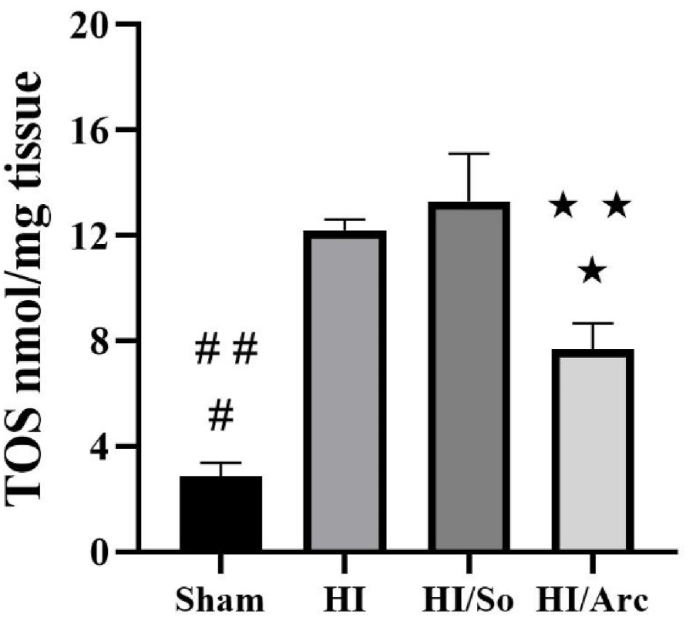
Total oxidant status (TOS) levels in the Sham, HI, HI/So, and HI/Arc groups (n = 8). Data are presented as mean ± SEM. #p < 0.0001 SHAM vs. HI and HI/So ##p = 0.0141 SHAM vs. HI/Arc ∗ p = 0.0103 HI/Arc vs. HI ∗∗p = 0.0030 HI/Arc vs. HI/So.

As indicated in Fig. 3, the mean TOS level in the Sham group (2.85 ± 0.51) was significantly lower than in the HI/So (13.29 ± 1.81) and HI groups (12.19 ± 0.42, p < 0.0001). Furthermore, the mean TOS level in the Sham group was significantly lower compared to the HI/Arctiin group (7.65 ± 0.99, p = 0.0141). The TOS level in the HI/Arctiin group was significantly lower than in the HI and HI/So groups (p = 0.0030).

**Fig. 4:**
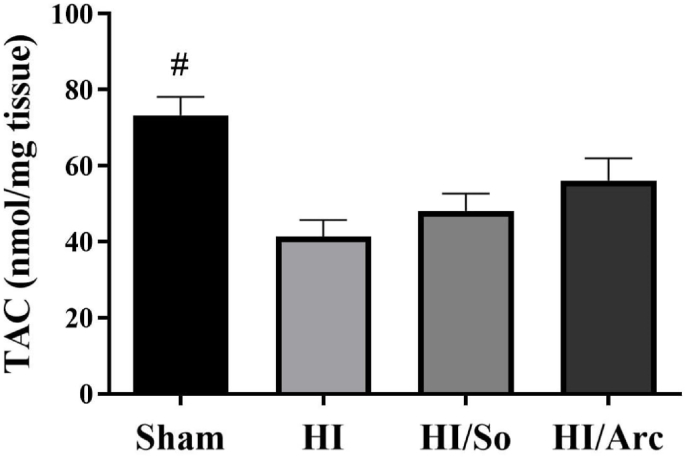
Total antioxidant capacity (TAC) levels in the Sham, HI, HI/So, and HI/Arc groups (n = 8). Data are presented as mean ± SEM. #P < 0.01 SHAM vs. HI and HI/So.

The mean TAC level in the Sham group (73.25 ± 4.85) was significantly higher compared to the HI (41.25 ± 4.45) and HI/So groups (48.12 ± 4.61, p < 0.01) (Fig. 4). Additionally, the mean TAC level in the HI/Arctiin group (56.12 ± 5.8) was significantly higher compared to the HI group.

### 3.4 Determination and comparison of BDNF levels in ischemic brain tissue

**Fig. 5:**
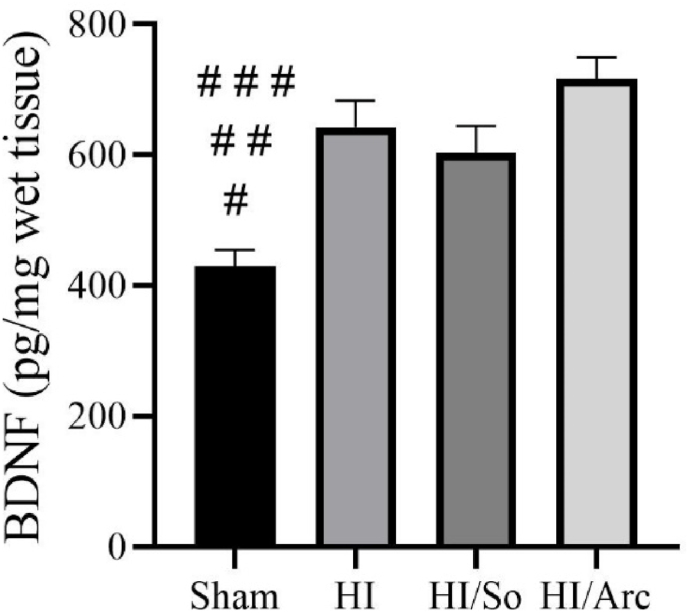
Brain-derived neurotrophic factor (BDNF) levels in the Sham, HI, HI/So, and HI/Arc groups (n = 8). Data are presented as mean ± SEM. #p < 0.0001 SHAM vs. HI/Arc # #p = 0.0119, HI/So with SHAM # # #p = 0.0015 SHAM vs. HI.

As shown in Fig. 5, the mean BDNF levels in the HI/Arctiin (716.8 ± 32.71), HI/So (602.75 ± 41.28), and HI (642.12 ± 41.58) groups were significantly higher compared to the Sham group (429.37 ± 25.72, p < 0.0001, p = 0.0015, p = 0.0119, respectively).

### 3.5 Comparison of infarct volume and brain edema

**Fig. 6:**
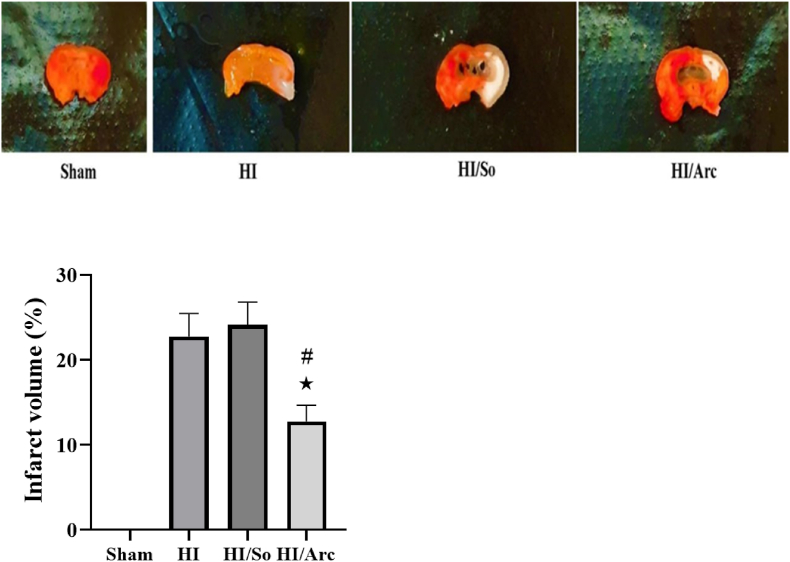
Infarct volume in the Sham, HI, HI/So, and HI/Arc groups (n = 8). Data are presented as mean ± SEM. ∗p = 0.0277 HI/Arc vs. HI # p = 0.0112, HI/Arc vs. HI/So.

The mean infarct volume percentage in the HI/Arctiin group (12.75 ± 1.91) was significantly lower compared to the HI group (22.75 ± 2.72, p = 0.0277) (Fig. 6). Additionally, the mean brain edema level in the HI/Arctiin group was significantly lower compared to the HI/So group (24.12 ± 2.67, p = 0.0112).

**Fig. 7:**
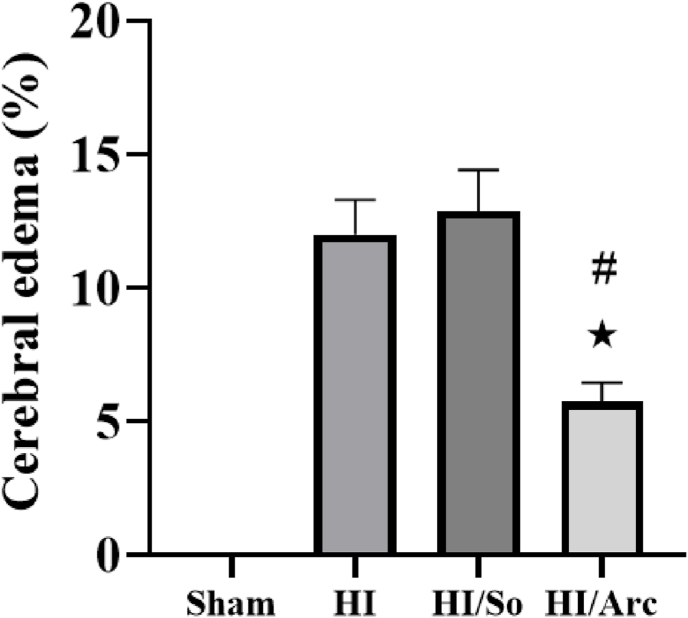
Cerebral edema levels in the Sham, HI, HI/So, and HI/Arc groups (n = 8). Data are presented as mean ± SEM. ∗p = 0.0055, HI/Arc vs. HI # p = 0.0017 HI/Arc vs. HI/So.

The mean brain edema percentage in the HI/Arctiin group (5.75 ± 0.70) was significantly lower compared to the HI group (12.00 ± 1.30, p = 0.0055) (Fig. 7). Furthermore, the edema level in the HI/Arctiin group was significantly lower than in the HI/So group (12.81 ± 1.55, p = 0.0017).

### 3.6 Behavioral tests

#### 3.6.1 Cliff avoidance test

**Fig. 8:**
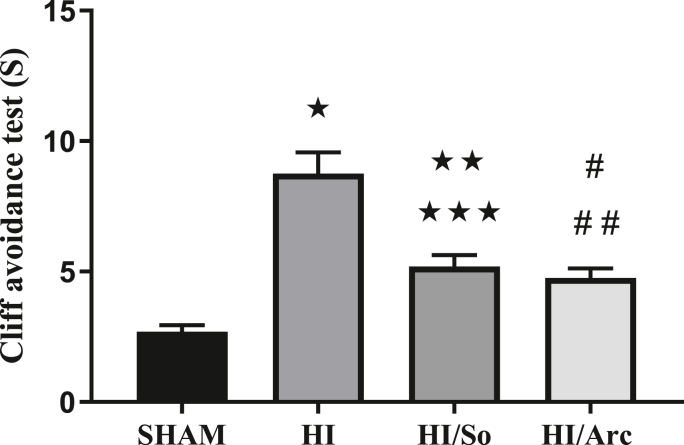
Comparison of the duration of the abyss avoidance reflex in the study group (n = 16). Data are presented as mean ± SEM. ∗p < 0.0001 HI vs. SHAM ##p = 0.0408 HI/Arc vs. SHAM ∗∗p < 0.0001 HI/So vs. SHAM ∗∗∗p = 0.0073 HI/So vs. HI # p < 0.0001 HI/Arc vs. HI.

As indicated in Fig. 8, the results from the cliff avoidance test revealed that the mean reflex duration in the HI group was significantly longer compared to the Sham group (p < 0.0001). The mean reflex duration in the HI/Arctiin group (4.75 ± 0.37) was significantly longer than in the Sham group (2.68 ± 0.25, p = 0.0408), but shorter than in the HI group (p < 0.0001). The mean reflex duration in the HI/So group (5.18 ± 0.44) was significantly longer compared to the Sham group (p < 0.0001), but shorter than in the HI group (8.75 ± 0.82, p = 0.0073).

#### 3.6.2 Negative geotaxis test

**Fig. 9:**
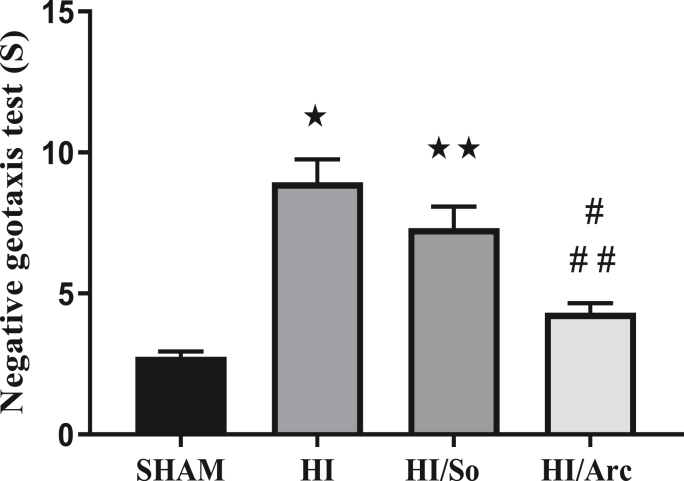
Comparison of the duration of the negative gravity reflex in the studied groups (n = 16). Data are presented as mean ± SEM. ∗p < 0.0001 HI vs. SHAM ∗∗p < 0.0001 HI/So vs. SHAM # #p < 0.0001 HI/Arc vs. HI # p = 0.0045 HI/Arc vs. HI/So.

As shown in Fig. 9, the mean reflex duration in the HI group (8.93 ± 0.82) was significantly longer compared to the Sham group (2.75 ± 0.19, p < 0.0001). The reflex duration in the HI/So group was also significantly longer than in the Sham group (p < 0.0001). Additionally, the mean reflex duration in the HI/Arctiin group (4.31 ± 0.33) was significantly shorter compared to the HI (p < 0.0001) and HI/So groups (p = 0.0045).

## 4 Discussion

Hypoxia-ischemia in human neonates is a major cause of brain damage and subsequent neurological disorders (Vannucci and Vannucci, 2005). Current treatments for reducing brain damage in neonates are limited, and most available drugs only mitigate damage rather than prevent it (Vannucci and Vannucci, 2005). In this context, arctiin presents itself as a new treatment option that could be used alone or in combination with other therapies. A more detailed investigation into the mechanisms by which arctiin affects Neuregulin 1 expression is essential. This effect might occur through the regulation of signaling pathways associated with transcription factors or directly impacting brain cells like glia and neurons. Additionally, the potential interactions between Neuregulin 1 and other genes involved in brain repair should not be overlooked. Further studies focusing on genetic and proteomic interactions could enhance our understanding of these mechanisms.

Natural products often possess anti-inflammatory and antioxidant properties. Numerous studies demonstrate that they can mitigate the destructive effects of inflammatory mediators and reactive oxygen species in various tissues (Shahcheraghi et al., 2023). Overall, our results indicate that arctiin can act as a potentially effective drug for treating brain injuries caused by HI in neonates. The increase in Neuregulin 1 expression highlights the importance of this gene in brain repair and the role of arctiin in enhancing this process. To confirm these findings and assess the long-term effects of arctiin, further studies using both animal and human models are necessary. Additionally, evaluating the side effects and safety of arctiin in neonates is also a critical aspect of future research. These findings lay the groundwork for developing new therapeutic approaches to reduce brain injuries caused by HI in human neonates. In the present study, hypoxic-ischemic induction resulted in increased inflammation, and treatment with Arctin reduced CRP levels, which serve as markers of the body's immune response to inflammation, after hypoxic-ischemic injury in the brain of the pups. Almeaqli et al. (2024) investigated the positive effects of Arctiin on cognitive performance and brain inflammation and oxidative stress in an Alzheimer's rat model (Almeaqli et al., 2024). The results showed that Arctiin improved behavior and brain structure while reducing brain inflammation. A study by Yuan et al. (2024) examined the effects of Arctin on asthma in mice. Researchers established an asthma model in mice and found that Arctiin reduced pathological lung changes, improved the progressive inflammatory response, enhanced lung function, and decreased oxidative stress (Yuan and Sun, 2024). In our study, Arctiin consumption by rat pups led to a reduction in CRP levels in the brain tissue of the Arctiin-treated group. Based on our results, it appears that Arctiin reduces inflammation induced by HI by decreasing the release of inflammatory factors, resulting in neuroprotective effects. In our study, the induction of hypoxia-ischemia resulted in increased oxidative stress, and treatment with Arctiin in rat pups led to a reduction in oxidative stress levels following hypoxic-ischemic brain injury. Chen et al. (2020) explored the effect of Arctiin on ischemia/reperfusion (I/R) cardiac injury in rats (Chen et al., 2020). The results showed that Arctiin reduced the levels of necroptosis-related proteins, oxidative stress, decreased necrosis and LDH release in hearts treated with I/R, and helped mitigate mitochondrial dysfunction in cardiomyocytes under H/R treatment (Chen et al., 2020). Consistent with our study, Arctiin consumption in rat pups increased TAC and decreased TOS, leading to reduced oxidative stress in brain tissue and decreased susceptibility to injury following HI. BDNF, a member of the neurotrophic family of growth factors, is responsible for the differentiation and survival of neurons in the brain (Fanaei et al., 2020). It also supports synaptic development, synaptogenesis, and synaptic stability (Fanaei et al., 2020). Research indicates that BDNF has protective properties against strokes, reducing cell death and infarct size while improving sensory-motor recovery after strokes (Bornavard and Fanaei, 2020; Khan et al., 2018). Furthermore, BDNF has antioxidant properties through positive regulation of superoxide dismutase and glutathione reductase expression, accompanied by reduced tyrosine nitration (Chen et al., 2017). In our study, hypoxic-ischemic induction led to neuronal cell death and brain tissue damage, and treatment with Arctiin in rat pups increased BDNF levels as an indicator of neuron health and function. A study by Wang et al. (2023) examined the laxative effects of Arctiin on functional constipation in mice (Wang et al., 2023). The results showed that Arctiin treatment increased levels of motilin and BDNF and reduced levels of NO and inflammation indicated positive effects of Arctiin in treating functional constipation and diminished colonic injury (Wang et al., 2023). Due to these positive effects, Arctiin is being considered as a therapeutic strategy for managing functional constipation (Wang et al., 2023). Our study aligns with this finding, showing that Arctiin increased BDNF levels, leading to enhanced neuron health and function in the brain tissue of rat pups. Xu et al. (2020) investigated the antidepressant mechanism of Arctigenin using a chronic unpredictable mild stress model in mice (Xu et al., 2020). The results showed that Arctigenin reduced immobility times in the tail suspension and forced swim tests, inhibited microglial activation and inflammatory responses, and suppressed the HMGB1/TLR4/NF-κB and TNF-α/TNFR1/NF-κB signaling pathways (Xu et al., 2020). Our behavioral test results showed that the immobility time in the Arctiin-treated group was shorter than that of the HI group, which is consistent with this study. Studies by Hidaka et al. (2023) suggested that arctigenin reduces diabetic complications, improves retinal edema, and increases the survival of retinal microvascular endothelial cells (Hidaka et al., 2023). Their results displayed oral arctigenin treatment reduces retinal edema and inflammation in mice (Hidaka et al., 2023). Our study's findings, regarding the protective effects of Arctiin against hypoxic-ischemic complications such as infarction and brain edema, align with this research. A study by Xia et al. (2021) examined the therapeutic effects of Arctiin on an amyotrophic lateral sclerosis model in mice (Xia et al., 2021). Arctiin was administered intraperitoneally daily for six weeks. The results showed that arctiin treatment reduced motor neuronal degeneration in the ventral horn of spinal cord and the reduction in grip strength in the arctiin group was less than control group (Xia et al., 2021). Our study's behavioral and histopathological findings also align with these results, showing significant neuroprotection after Arctiin treatment.

The molecular and biochemical improvements elicited by Arctiin translated into significant histological neuroprotection, as evidenced by the marked reduction in both cerebral infarct volume and brain edema. The decrease in brain edema is a direct correlate of diminished neuroinflammation and blood-brain barrier disruption, processes that were mitigated by Arctiin's suppression of CRP and oxidative stress. Similarly, the significant reduction in infarct area, visualized by TTC staining, underscores the compound's efficacy in limiting the progression of ischemic cell death. This structural preservation is likely the combined result of attenuated oxidative damage, quelled inflammatory cascades, and the enhanced trophic support signaled by increased BDNF and restored NRG1 expression. Ultimately, these tangible histological benefits provide the basis for the observed functional recovery, forming a coherent pathway from molecular intervention to tissue integrity and improved neurobehavioral outcomes.

This study makes several distinct contributions to the field of neonatal neuroprotection. Firstly, it provides novel evidence that the neuroprotective efficacy of Arctiin in a neonatal hypoxic-ischemic brain injury model is mechanistically linked to the restoration of NRG-1 expression, a key modulator of neuroglial function and repair previously unexplored in this therapeutic context (Ryu et al., 2019). Secondly, it demonstrates that post-injury oral administration of Arctiin successfully integrates a multi-target strategy, simultaneously mitigating oxidative stress and neuroinflammation while upregulating protective trophic factors (BDNF and NRG-1), which translates into measurable histological and functional recovery. By establishing a coherent pathway from molecular intervention to improved sensorimotor outcomes, our work positions Arctiin not merely as another antioxidant compound, but as a promising multi-faceted candidate for adjunctive therapy, addressing the critical need for safe, translatable pharmacological strategies to complement standard care in neonatal encephalopathy.

A limitation of this study is the use of a single dose of Arctiin (60 mg/kg). This dosage was selected based on its proven efficacy in mitigating oxidative injury and inflammation in a prior study in wistar rats (Liu et al., 2021) (Liu et al., 2021). While this established dose allowed us to effectively demonstrate Arctiin's neuroprotective potential, our experimental design does not provide information on a dose-response relationship or define the optimal therapeutic dosage for HIBD. Consequently, the observed benefits represent a specific effect at this concentration. Future studies incorporating multiple dose levels are essential to determine the minimum effective dose, the dose for maximal efficacy, and to thoroughly assess the therapeutic window for Arctiin in the context of neonatal brain injury.

## 5 Conclusion

In summary, this study demonstrates that post-injury oral administration of Arctiin provides significant neuroprotection in a neonatal rat model of hypoxic-ischemic brain damage. The key findings indicate that Arctiin's efficacy is mediated by a multi-targeted action: reducing oxidative stress and inflammation, enhancing antioxidant capacity, and critically, restoring the expression of the neuroprotective gene Neuregulin-1 while elevating BDNF levels. These molecular and biochemical benefits translated into tangible histological and functional improvements, including reduced cerebral infarct volume, attenuated edema, and enhanced sensorimotor performance. While these results are promising, our study has several limitations that define clear avenues for future research. First, the investigation employed a single dose and a fixed treatment duration; future studies should establish a dose-response relationship and optimal therapeutic window. Second, the long-term neurocognitive outcomes and potential side effects of Arctiin treatment remain to be evaluated. Third, the precise upstream signaling pathways through which Arctiin modulates NRG-1 expression and converges on anti-inflammatory and antioxidant effects require elucidation, potentially involving detailed investigation of the Nrf2 and NF-κB pathways. Addressing these limitations in subsequent work will be crucial to fully assess the translational potential of Arctiin as a adjunctive therapy for neonatal hypoxic-ischemic encephalopathy.

## Disclosure statement

All authors declare that they have no actual or potential conflict of interest, including any financial, personal, or other relationships with other people or organizations.

## Ethics approval statement

The study was approved by Ethics Committee of Zahedan University of Medical Sciences (ethical code: IR.ZAUMS.AEC.1402.002).

## Author contributions

Hamed Fanaei designed the study. Skandar Babak, Hamed Fanaei, and Tahereh Safari carried out the experiments. Hamed Fanaei and Skandar Babak analyzed the data. Hamed Fanaei and Skandar Babak wrote the manuscript.

## Funding sources

Financial support for the study was conducted by the Office of Vice-President for Research and Information Technology of 10.13039/501100004847Zahedan University of Medical Sciences (3786).

## Declaration of competing interest

The authors declare that they have no known competing financial interests or personal relationships that could have appeared to influence the work reported in this paper.

## Data Availability

Data will be made available on request.

## References

[bib1] Ahmed S. (2025). Antioxidant assays in phytonutrient research: translating laboratory innovations into practical applications. PHYTONutrients.

[bib2] Al-Jaber H.I., Shakya A.K., Al-Qudah M.A., Barhoumi L.M., Abu-Sal H.E., Hasan H.S. (2024). Piceatannol, a comprehensive review of health perspectives and pharmacological aspects. Arab. J. Chem..

[bib3] Almeaqli M.T., Alaidaa Y., Alnajjar F.M., Al Shararh A.S., Alharbi D.S., Almslmani Y.I. (2024). Therapeutic effects of arctiin on alzheimer's disease-like model in rats by reducing oxidative stress, inflammasomes and fibrosis. Curr. Alzheimer Res..

[bib4] Bornavard M., Fanaei H. (2020). Morphine consumption during pregnancy exacerbates neonatal hypoxia-ischemia injury in rats.

[bib5] Bornavard M., Fanaei H., Mirshekar M.A., Farajian Mashhadi F., Atashpanjeh A. (2020). Morphine consumption during pregnancy exacerbates neonatal hypoxia-ischemia injury in rats. Int. J. Dev. Neurosci. : off. J. Int. Soc. Dev. Neurosci..

[bib6] Ceri A., Ozdemir F.M.A. (2025). Hypoxic-ischemic encephalopathy in neonates: emerging challenges and future directions. Maternal Children’s Health.

[bib7] Chen X., Cheang W. (2024). Protective effects of piceatannol on cardiovascular diseases. PHYTONutrients.

[bib8] Chen S.-D., Wu C.-L., Hwang W.-C., Yang D.-I. (2017). More insight into BDNF against neurodegeneration: anti-apoptosis, anti-oxidation, and suppression of autophagy. Int. J. Mol. Sci..

[bib9] Chen H., Tang L.J., Tu H., Zhou Y.J., Li N.S., Luo X.J. (2020). Arctiin protects rat heart against ischemia/reperfusion injury via a mechanism involving reduction of necroptosis. Eur. J. Pharmacol..

[bib10] Fanaei H., Riki F., Khayat S., Bornavard M. (2020). Brain-derived neurotrophic factor and nerve growth factor concentrations in maternal and umbilical cord blood of opium-addicted mothers. Int. J. Dev. Neurosci..

[bib11] Gao Q., Yang M., Zuo Z. (2018). Overview of the anti-inflammatory effects, pharmacokinetic properties and clinical efficacies of arctigenin and arctiin from Arctium lappa L. Acta Pharmacol. Sin..

[bib12] Hidaka Y., Nakamura S., Nishinaka A., Takajo Y., Inamasu S., Yomoda S. (2023). Arctigenin prevents retinal edema in a murine retinal vein occlusion model. Biol. Pharmaceut. Bull..

[bib13] Khan H., Amin S., Patel S. (2018). Targeting BDNF modulation by plant glycosides as a novel therapeutic strategy in the treatment of depression. Life Sci..

[bib14] Khayat S., Fanaei H., Lakzaee N. (2023). Effects of prenatal Mobile phone radiation exposure on MMP9 expression: implications for inflammation, oxidative stress, and sensory-motor impairment after neonatal hypoxia- ischemia in rats. Toxicol. Rep..

[bib15] Lan X.-B., Wang Q., Yang J.-M., Ma L., Zhang W.-J., Zheng P. (2019). Neuroprotective effect of vanillin on hypoxic-ischemic brain damage in neonatal rats. Biomed. Pharmacother..

[bib16] Liu X., Wang J., Dou P., Zhang X., Ran X., Liu L. (2021). The ameliorative effects of arctiin and arctigenin on the oxidative injury of lung induced by silica via TLR-4/NLRP3/TGF-β signaling pathway. Oxid. Med. Cell. Longev..

[bib17] Mohsenpour H., Pesce M., Patruno A., Bahrami A., Pour P.M., Farzaei M.H. (2021). A review of plant extracts and plant-derived natural compounds in the prevention/treatment of neonatal hypoxic-ischemic brain injury. Int. J. Mol. Sci..

[bib18] Reyes-Corral M., Sola-Idígora N., de la Puerta R., Montaner J., Ybot-González P. (2021). Nutraceuticals in the prevention of neonatal hypoxia–ischemia: a comprehensive review of their neuroprotective properties, mechanisms of action and future directions. Int. J. Mol. Sci..

[bib19] Ryu S., Lee J.M., Bae C.A., Moon C.E., Cho K.O. (2019). Therapeutic efficacy of neuregulin 1-expressing human adipose-derived mesenchymal stem cells for ischemic stroke. PLoS One.

[bib20] Sabir H. (2025). Neuroprotective therapies for neonatal hypoxic-ischemic brain injury – a contemporary update. Semin. Perinatol..

[bib21] Shahcheraghi S.H., Salemi F., Small S., Syed S., Salari F., Alam W. (2023). Resveratrol regulates inflammation and improves oxidative stress via Nrf2 signaling pathway: therapeutic and biotechnological prospects. Phytother Res..

[bib22] Shyam M., Sabina E.P. (2024). Harnessing the power of Arctium lappa root: a review of its pharmacological properties and therapeutic applications. Nat. Product. Bioprospect..

[bib23] Vannucci R.C., Vannucci S.J. (2005). Perinatal hypoxic-ischemic brain damage: evolution of an animal model. Dev. Neurosci..

[bib24] Wang Y., Jiang H., Wang L., Gan H., Xiao X., Huang L. (2023). Arctiin alleviates functional constipation by enhancing intestinal motility in mice. Exp. Ther. Med..

[bib25] Xia L., Hua L., Sun S., Yang X., Chen X., Chen S. (2021). 2021 IEEE International Ultrasonics Symposium (IUS).

[bib26] Xu X., Piao H.N., Aosai F., Zeng X.Y., Cheng J.H., Cui Y.X. (2020). Arctigenin protects against depression by inhibiting microglial activation and neuroinflammation via HMGB1/TLR4/NF-κB and TNF-α/TNFR1/NF-κB pathways. Br. J. Pharmacol..

[bib27] Yosri N., Alsharif S.M., Xiao J., Musharraf S.G., Zhao C., Saeed A. (2023). Arctium lappa (Burdock): insights from ethnopharmacology potential, chemical constituents, clinical studies, pharmacological utility and nanomedicine. Biomed. Pharmacother..

[bib28] Yuan L., Sun C. (2024). The protective effects of arctiin in asthma by attenuating airway inflammation and inhibiting p38/NF-κB signaling. Aging.

